# Investigation of human infection with H5N6 avian influenza cases in Sichuan Province from 2014 to 2024: a retrospective study

**DOI:** 10.3389/fpubh.2025.1603158

**Published:** 2025-06-06

**Authors:** Lijun Zhou, Zhirui Li, Xingyu Zhou, Lin Zhao, Huanwen Peng, Xunbo Du, Jianping Yang, Fengmiao Hu, Shuang Dong, Baisong Li, Guidan Liu, Hongyu Tang, Xiao Lei, Xiaojuan Wang, Shunning Zhao, Ping Zhou, Heng Yuan, Chongkun Xiao

**Affiliations:** ^1^Department of Disease Control and Prevention, Sichuan Provincial Center for Disease Control and Prevention, Chengdu, China; ^2^Nanchong Center for Disease Control and Prevention, Nanchong, China; ^3^Dazhou Center for Disease Control and Prevention, Nanchong, China; ^4^Chengdu Center for Disease Control and Prevention, Chengdu, China; ^5^Bazhong Center for Disease Control and Prevention, Bazhong, China; ^6^Chongqing Center for Disease Control and Prevention, Chongqing, China; ^7^Zigong Center for Disease Control and Prevention, Zigong, China; ^8^Deyang Center for Disease Control and Prevention, Deyang, China; ^9^Leshan Municipal Center for Disease Control and Prevention, Leshan, China; ^10^Luzhou Center for Disease Control and Prevention, Luzhou, China; ^11^Ziyang Center for Disease Control and Prevention, Ziyang, China; ^12^Yibin Center for Disease Control and Prevention, Yibin, China

**Keywords:** influenza, epidemiology, avian, H5N6, poultry diseases

## Abstract

**Objective:**

The objective is to examine the epidemiology and clinical features of human cases infected with H5N6 avian influenza in Sichuan Province from 2014 to 2024, and to offer guidance for the prevention and management of human infections with H5N6 avian influenza.

**Methods:**

Epidemiological survey reports of H5N6 avian influenza cases in Sichuan Province from 2014 to 2024 were compiled, and the epidemiological context and characteristics of 16 human cases infected with H5N6 avian influenza in the province were summarized and analyzed using descriptive epidemiological methods.

**Results:**

From 2014, when the initial human case ofH5N6 infection was documented in Sichuan Province, to 2024, there have been 16 human cases of H5N6 avian influenza in the region, resulting in 12 fatalities and a case fatality rate of 75%. The instances were predominantly located in the Chengdu Plain, eastern Sichuan, and southern Sichuan.

**Conclusion:**

Human instances of H5N6 avian influenza in Sichuan Province exhibit no discernible periodicity, and entail significant fatality rates. It is essential to enhance the early diagnosis and treatment of avian influenza cases in medical facilities, prioritize farmers with preexisting conditions who have been in contact with deceased poultry, conduct influenza virus testing promptly, and administer antiviral medications at the earliest opportunity. Simultaneously, we must effectively engage in public awareness and education for the populace, manage poultry scientifically, and prevent direct contact with deceased poultries.

## 1 Introduction

The initial human instance of H5N6 influenza was documented in Sichuan Province in 2014, with a resurgence occurring in 2021. By December 31, 2024, there had been 16 documented cases of human infection with H5N6 avian influenza (1). Avian influenza viruses (AIVs) are zoonotic and provide a continual public health risk globally (2). The World Organization for Animal Health characterizes avian influenza as a highly transmissible illness resulting from several subtypes that persistently develop, impacting poultry, avian species, mammals, and, on occasion, humans (3).

The influenza virus is a single-stranded Ribonucleic Acid (RNA) virus categorized into types A, B, C, and D based on antigenic variations in the matrix protein and nucleoprotein (4). The avian influenza virus is classified as type A (5). The avian influenza virus mostly affects avians and sometimes humans. The incubation time for human infection with avian influenza typically ranges from 2 to 5 days, but the incubation period for H5N6 spans from 1 to 13 days, averaging 4.3 days (6). H5N6 avian influenza is a severe respiratory illness. The severity of avian influenza virus infection in humans can vary from asymptomatic or moderate flu-like symptoms to severe respiratory infections, including pneumonia, multiple organ failure, or death (7, 8). The severity of the sickness is contingent upon the subtype of the avian influenza virus responsible for the infection and the physical state of the afflicted individual (9).

While the majority of documented human H5N6 cases have transpired in China, isolated instances of H5N6 viruses have also been detected in poultry and wild avifauna throughout other Southeast Asian nations, including Vietnam and Laos, underscoring the regional dissemination of the virus (10, 11). The H5N6 virus emerged from the reassortment of H5N1 and H5N2 with H6N6 viruses. The hemagglutinin (HA) gene is classified under the H5 evolutionary lineage 2.3.4.4, although its neuraminidase (NA) gene is derived from the H6N6 strain commonly seen in Asian poultry (12, 13). The preliminary reassortment event likely transpired in ducks, who serve as primary hosts for viral amalgamation owing to their vulnerability to various influenza subtypes (14). Following the introduction of the H5N6 virus, genetic reassortment transpired with low pathogenic avian influenza viruses, leading to the selection and evolution of dominant genotypes (G1, G2, G1.1, G1.2) (13). The reassortment events increased the adaptability and transmissibility of the H5N6 virus. Recent research in Sichuan Province identified new triple reassortant H5N6 strains, including genes from H5N8, H6N6, and H9N2, underscoring continuous genetic evolution (15, 16). Moreover, H5N6 viruses have been identified in animals including pigs, cats, and wild birds, suggesting their capacity for interspecies transmission (17).

**Figure 1:**
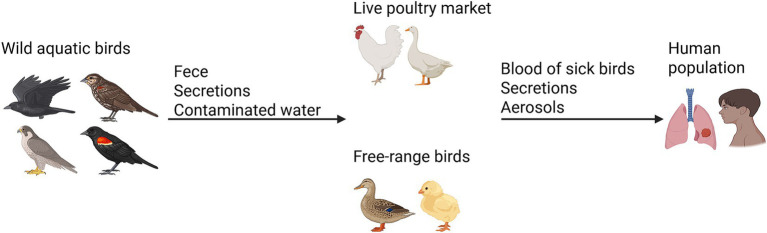
A conceptual framework for the dissemination of H5N6.

**Figure 2:**
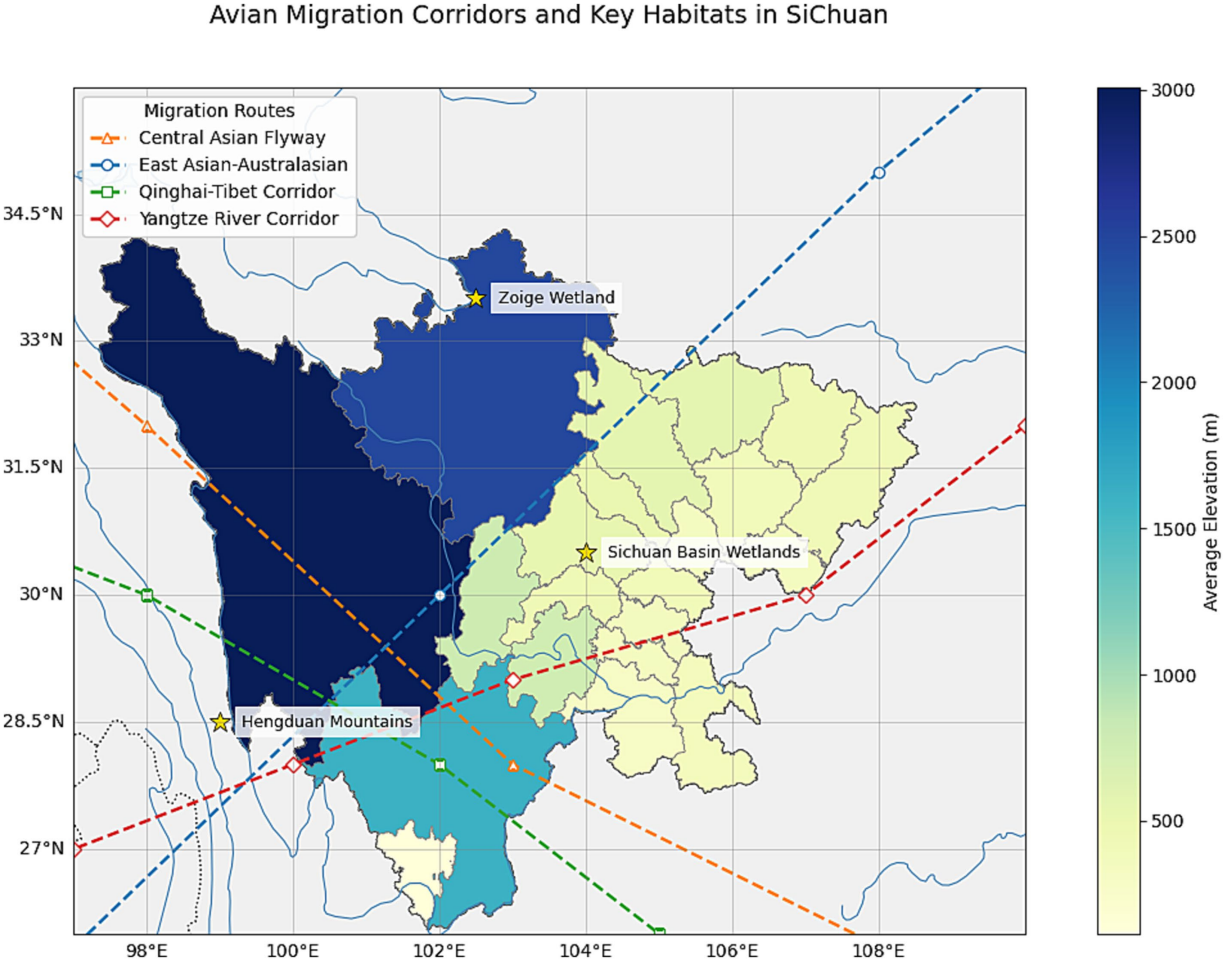
Key time intervals in H5N6 cases, Sichuan Province, 2014–2024.

The H5N6 avian influenza virus continues to represent a significant risk to both poultry and humans (18). A conceptual framework for the dissemination of H5N6 is illustrated in Figure 1. Current reports indicate that, as of April 30, 2025, all documented H5N6 infections originated from China, with the exception of a single case identified in Laos (19). Sichuan Province is the inaugural location where H5N6 was identified globally. This can be elucidated from the following perspectives: (1) Ecological characteristics: Sichuan Province features a diverse terrain with basins, mountains, and plateaus. The climatic classifications encompass subtropical to cold zones, primarily comprising subtropical humid and high-cold climates. This habitat may facilitate the proliferation and dissemination of avian influenza viruses; Sichuan serves as a crucial water supply in the upper sections of the Yangtze and Yellow Rivers, boasting abundant wetland and forest resources that attract numerous migratory birds. Nevertheless, migratory birds may have viruses, heightening the danger of cross-regional transmission of avian influenza (20). (2) Agricultural attributes: Sichuan is a significant rice cultivation region, necessitating substantial water resources. The mixed farming model of rice cultivation and poultry is prevalent, enhancing the likelihood of interaction between poultry and people; live poultry markets and poultry farming are widespread in rural Sichuan, with mixed farming practices present in certain regions. This approach elevates the likelihood of viral reassortment in avian species and its transfer to humans (21). (3) Population characteristics: Sichuan possesses a significant rural demographic, with rural inhabitants exhibiting a lack of awareness regarding avian influenza, hence enhancing the danger of outbreaks (22). (4) Additional factors: AIVs are primarily spread among birds, poultry, and people via migratory bird movements, poultry farms, and transactions in live poultry markets (22, 23). Wild birds serve as natural reservoirs for avian influenza and may disseminate the genetic material of all influenza A virus strains (24). China acts as a significant transit hub for worldwide migratory avifauna. China has three major international migration routes, with two traversing Sichuan Province: the Central Asian Migration Route and the East Asian-Australasian Migration Route (25–27). Two of the three migration routes in China, namely the central and western routes, traverse the Sichuan Basin, where the East Asian-Australasian Migration Route intersects with the Central Asian Migration Route (28, 29). Sichuan Province has emerged as a significant stopover location for several migrating birds, owing to its elevated wetland habitats, diverse wetland ecosystems, and vertical migration pathways (Figure 2). Consequently, the species variety of wild avifauna is greater (30, 31). Migratory birds typically migrate from March to May during spring and from September to November in fall (32, 33).

The primary risk factors for avian influenza infection in poultry farms are wild birds and associated environments, unprotected water sources, and vulnerable animals (34, 35). Fecal-oral transmission constitutes the primary pathway for avian influenza dissemination in poultry, with the virus expelled in elevated titers via feces (36, 37). In regions where poultry farming land coincides with migratory bird pathways, there exists a conduit for the transmission and interchange of viruses between domesticated fowl and wild avians (38). A significant flow of viruses transpires between poultry and adjacent wild birds, potentially resulting in antigenic drift and antigenic shift, finally culminating in the modification of the avian influenza virus (39). The altered novel virus may possess an enhanced capacity to infect animals, including humans. While the transmission pathway and infection mechanism of the avian influenza virus in mammals remain ambiguous, data indicates that several mammalian species have been infected with the virus (40, 41). Future hazards of avian influenza pandemics may arise from frequent genetic recombination and interspecies transmission, necessitating more effective measures to mitigate the risk posed by AIVs (2).

The ecological and geomorphological variety, agricultural structural attributes, and demographic features of Sichuan Province have collectively fostered an environment conducive to the proliferation of avian influenza viruses. This publication analyses and categorizes human cases of H5N6 avian influenza identified in Sichuan Province throughout the years to serve as a reference for the prevention and management of human avian influenza. We further suggested evidence-based solutions to enhance the surveillance system, particularly at high-frequency interaction places between people and poultry, and to priorities preventative interventions for high-risk groups.

## 2 Methods

### 2.1 Patients

The cases were individuals infected with the influenza A(H5N6) virus reported in Sichuan Province from January 1, 2014, to April 30, 2025. The case data were obtained from the infectious illness monitoring and reporting system and case survey reports of the China Disease Prevention and Control Information System. The primary information encompassed age, gender, employment, domicile, onset time, hospitalization duration, medical history, poultry exposure history, specimen collection, and laboratory test findings etc. The data were derived from epidemiological surveys and laboratory testing performed by medically trained investigators and physicians, after patient consent. Sichuan Province has implemented a sentinel hospital monitoring network including all cities since 2009. Each sentinel hospital gathers 10–40 specimens of influenza-like cases weekly and does nucleic acid testing for the influenza virus. No mild cases of H5N6 avian influenza have been identified to yet.

### 2.2 Environmental monitoring

According to the Technical Guidelines for the Prevention and Control of Influenza of Human Infection of Animal Origin, all cities (21) within the province are implementing external environmental surveillance for avian influenza. Two or more locations are designated, encompassing live poultry market, Family poultry farm, Poultry processing plants, Wild migratory bird habitat, with monitoring conducted monthly (42). A minimum of 10 specimens are gathered monthly, totaling 120 specimens each city year.

### 2.3 Research methods

Real-time Quantitative PCR was employed to identify influenza virus nucleic acid in environmental samples from confirmed patients and probable exposure locations. The case definition and monitoring techniques have remained unchanged over the past decade. A case is characterized by the isolation of the avian influenza virus from an individual’s respiratory secretions or other pertinent materials, or the detection of the virus using nucleic acid testing or deep sequencing. In Sichuan Province, case confirmation mostly relies on positive nucleic acid test findings and associated clinical signs. Close contacts were identified as those who interacted with cases and personnel who failed to implement adequate protective measures throughout the diagnosis and treatment of cases; this includes individuals who had close contact with cases from 1 day prior to the start of symptoms to the initiation of isolation. We gathered patients’ medical records and performed verification, monitoring, and medical assessment in compliance with the Technical Guidelines for the Prevention and Control of Animal-Origin Influenza in Human Infection. Close contacts were monitored according to the comprehensive range of activities from the day preceding the commencement of the case until isolation treatment or death. Close contacts encompass: medical personnel who inadequately safeguarded themselves while diagnosing and treating suspected, clinically diagnosed, or confirmed cases, as well as other individuals who attended to these cases; individuals residing with or having close interactions with suspected, clinically diagnosed, or confirmed cases from 1 day prior to the onset of the illness until isolation treatment or death; and additional individuals requiring management following an on-site investigation.

In order to more accurately represent the central trend and variations, we employed box plots to emphasize the distribution of three distinct time periods: (a) time from onset to hospitalization, (b) time from hospitalization to commencement of treatment, and (c) time from treatment to outcome.

### 2.4 Statistical analysis

The monitoring data were compiled using Excel 2019, and the descriptive epidemiological approach was employed to examine the outcomes of human infections with H5N6 avian influenza. The statistical analysis software employed was R version 4.2.1. Owing to the exceedingly limited sample size of our study (e.g., survival group *n* = 4), the *p* value may not accurately represent the genuine difference; hence, the effect size is illustrated through the confidence interval. Continuous variables are expressed as mean ± SD (95% CI), with group differences indicated by the mean difference via Welch’s t test; categorical variables are represented as proportions % (95% CI), with group differences denoted by the proportion difference using the Newcombe-Wilson method. Data visualization and visuals were created using the VS Code editor and the Python programming language, using relevant libraries like Matplotlib 3.5.1 and Seaborn 0.11.2.

## 3 Results

### 3.1 Demographic distribution

Table 1 presents the fundamental details of the 16 instances. The male-to-female ratio among the patients was 1:1, and the case fatality rate was 75%; the median age was 54.5 years (IQR: 27–75); the occupational distribution predominantly consisted of farmers, accounting for 12 cases (75%). Of the 16 patients, 12 (75%) had preexisting conditions, while 4 cases (25%) were previously healthy. The case fatality rate for patients with underlying conditions was 83.33%, above that of individuals without such conditions, which was 50.00%.

**Table 1:** Basic information on human infections of H5N6 avian influenza in Sichuan Province from 2014 to 2024.

Characteristics	Deceased (*n* = 12) (95% CI)	Survived (*n* = 4) (95% CI)	Between-group difference (95% CI)
Age (year, mean ± SD)	52.8 ± 15.7 (42.3–63.2)	55.8 ± 7.9 (44.9–66.6)	Δ-3.0 (−20.1–14.1)
Gender n (%)			
Female	3 (25.0%, 5.5–57.2%)	2 (50.0%, 6.7–93.3%)	Δ-25.0% (−64.1–14.1%)
Male	9 (75.0%, 42.8–94.5%)	2 (50.0%, 6.7–93.3%)	Δ25.0% (−14.1–64.1%)
Occupation n (%)			
Farmer	9 (75.0%, 42.8–94.5%)	3 (75.0%, 19.4–99.4%)	Δ0.0% (−48.1–48.1%)
Others	3 (25.0%, 5.5–57.2%)	1 (25.0%, 0.6–80.6%)	Δ0.0% (−48.1–48.1%)
Time Intervals (days, mean ± SD)			
Onset to hospital admission	5.4 ± 4.3 (2.6–8.3)	3.3 ± 1.5 (0.9–5.6)	Δ2.1 (−2.3–6.5)
Hospital admission to ICU entry	1.6 ± 1.2 (0.8–2.4)	3.0 ± 2.2 (0.0–6.5)	*Δ*-1.4 (−3.9–1.1)
Onset to diagnosis	11.2 ± 6.5 (6.9–15.4)	9.5 ± 4.4 (2.7–16.3)	Δ1.7 (−6.1–9.5)
Hospital admission to initiation of antiviral treatment	2.1 ± 3.0 (0.1–4.1)	1.3 ± 0.5 (0.0–2.5)	Δ0.8 (−1.8–3.4)
Interventions			
ECMO* use (%)	33.3 (9.9–65.1)	25.0 (0.6–80.6)	Δ8.3 (−34.1–50.7)
Antiviral treatment use (%)	83.3 (51.6–97.9)	100.0 (39.8–100.0)	Δ-16.7 (−44.4–11.0)
Exposure history			
Contact with sick/dead poultry (%)	58.3 (27.7–84.8)	75.0% (19.4–99.4%)	Δ-16.7% (−54.1–20.7%)

* ECMO: Extracorporeal Membrane Oxygenation.

### 3.2 Geographical allocation

**Figure 3:**
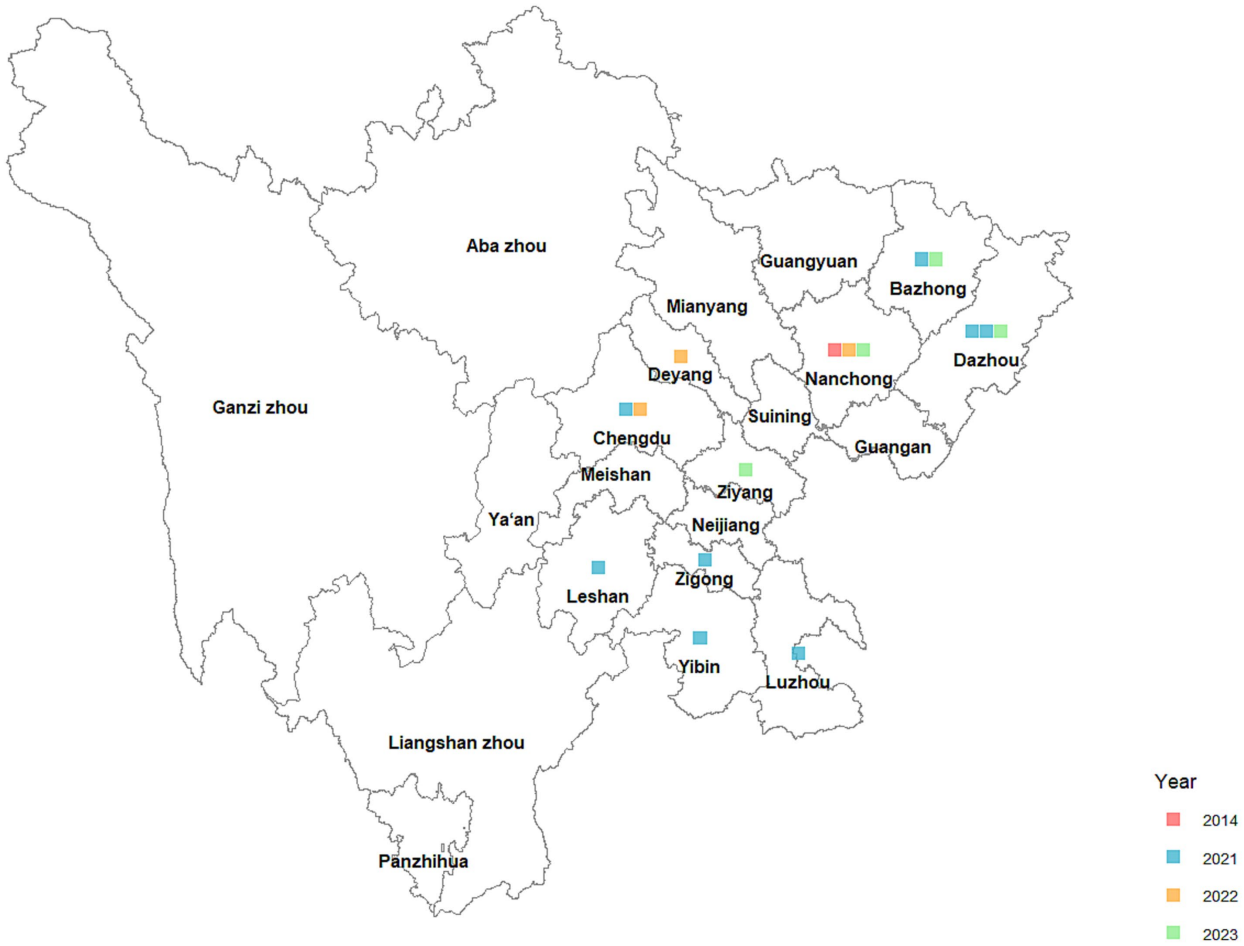
Geographical distribution of human infections with H5N6 avian influenza in Sichuan Province, 2014–2024.

Figure 3 illustrates 16 instances of human infection with H5N6 avian influenza identified in Sichuan Province between 2014 and 2024. The instances were predominantly located in the Chengdu Plain, as well as eastern and southern Sichuan. Nanchong and Dazhou had the highest incidence, with 3 instances apiece; followed by Chengdu and Bazhong, each with 2 cases; and Deyang, Leshan, Luzhou, Yibin, Ziyang, and Zigong, each with 1 case.

### 3.3 Onset and treatment

**Figure 4:**
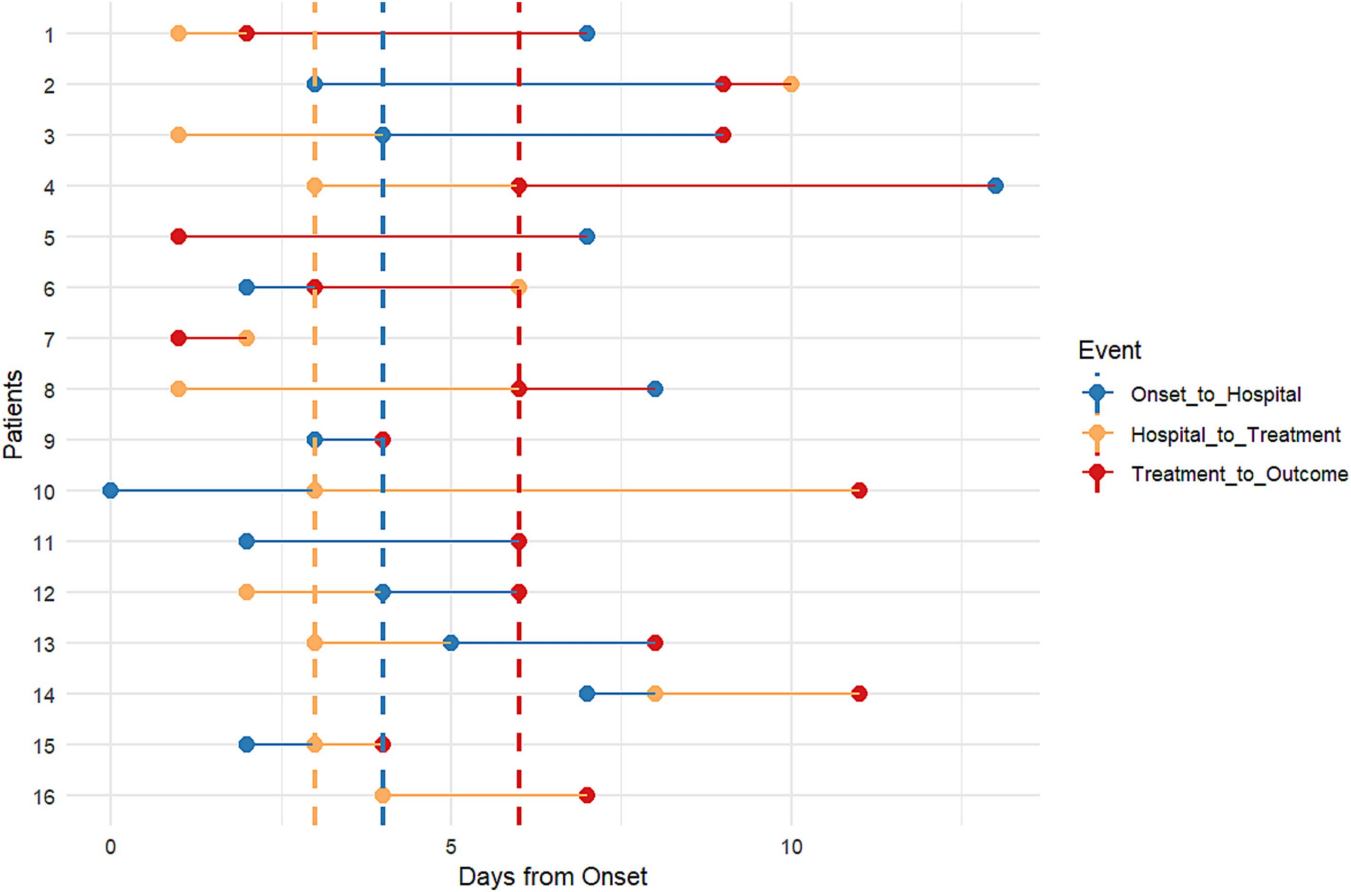
Avian migration corridors and key habitats in Sichuan.

**Figure 5:**
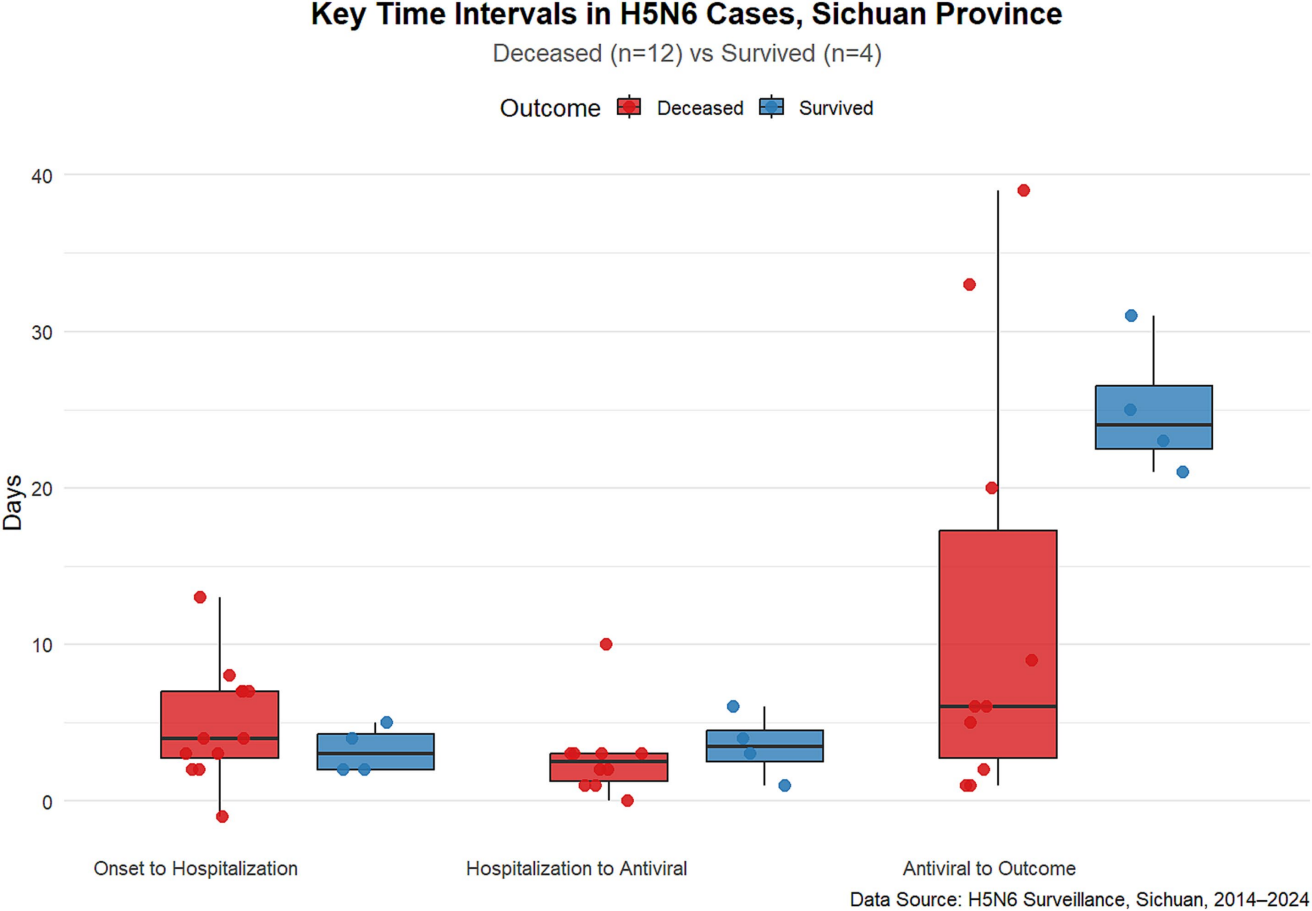
Timeline of “Onset to Hospital,” “Hospital to Treatment,” “Treatment to Outcome” of human cases of avian influenza H5N6 in Sichuan Province, 2014–2024.

Figures 4, 5 illustrate the timings of the three critical nodes: “onset to hospitalization,” “hospitalization to treatment,” and “treatment to outcome” for the 16 patients, along with the disparities in the distribution of these three indicators between the mortality group and the survival group. The median duration from beginning to hospitalization was 4 days (range: 0–13 days), suggesting that while some patients were treated promptly, others had significant delays. The median duration from hospitalization to the initiation of antiviral medication was 3 days (range: 1–10 days), indicating a potential delay in commencing treatment post-hospitalization. The median period from therapy to result was 6 days (range: 1–11 days), reflecting variability in illness regression.

The findings on the duration from beginning to hospitalization indicated that the median disparity between the mortality group and the survival group was minimal, with a significant overlap in distribution, predominantly concentrated between 5 to 10 days. The analysis of the duration from hospitalization to the initiation of antiviral therapy indicated that the survival cohort commenced treatment more uniformly and somewhat sooner, whereas the timing of treatment initiation in the mortality cohort exhibited greater variability, with notable delays in certain instances. The duration from antiviral therapy to outcome was shorter in the mortality group, suggesting fast disease progression. The duration from antiviral therapy to discharge/recovery in the survival cohort was comparatively prolonged, aligning with the clinical recovery trajectory. Our findings indicated a tendency of differences between the mortality group and the survival group; however, none achieved statistical significance, likely attributable to the limited sample size impacting statistical power. The occurrence of delayed treatment and subsequent quick mortality in the deceased cohort is noteworthy and will be further examined in a larger sample in the future.

### 3.4 Poultry exposure

Table 2 presents the poultry exposure history associated with the patients. Following epidemiological research and laboratory analysis, 10 of the 16 patients exhibited a history of contact with deceased poultry, representing 62.5%; 12 cases, or 75.00%, tested positive for H5N6 or H5 subtypes in chicken specimens at their residences. A total of 203 close connections of the 16 patients were identified, and all close contacts received health monitoring for a minimum of 10 days. No anomalies were identified during the health monitoring period, and no influenza virus was discovered in respiratory specimens at the commencement and conclusion of the monitoring.

**Table 2:** Environmental exposure history of H5N6 avian influenza cases in Sichuan Province from 2014 to 2024.

Year	Region	Live Poultry Market Exposure	Positive specimens detected in the market	Diseased or dead birds in and around your residence	Exposure to diseased or dead birds	Consumption of diseased or dead birds	Types of sick and dead birds	Positive avian specimens in residential environment
2014	Nanchong^1^	Yes	No	Yes	Yes	Yes	Chicken, Duck, Goose	Yes
2021	Chengdu^1^	Yes	Yes	No	No	No	—	No
2021	Bazhong^1^	No	Untested	Yes	Yes	No	Chicken, Duck, Goose	Yes
2021	Dazhou^1^	Yes	Yes#	Yes	Yes	Yes	Chicken, Duck	Yes#
2021	Dazhou^2^	Yes	Yes	Yes	Yes	Unknown	Chicken, Duck	Yes#
2021	Yibin	Yes	Yes	Yes	Yes	Yes	Chicken	Yes#
2021	Zigong	Unknown	Untested	Yes	Yes	Yes	Chicken, Duck	Yes
2021	Leshan	Unknown	Untested	Yes	Yes	Yes	Chicken	Yes
2021	Luzhou	Unknown	Yes	Yes	Yes	Yes	Chicken, Duck	Yes
2022	Nanchong^2^	No	No	No	No	No	—	Yes
2022	Chengdu^2^	Yes	Yes	No	No	No	—	No
2022	Deyang	No	No	No	Yes	No	Chicken	No
2023	Nanchong^3^	Yes	Yes	Yes	Yes	No	Chicken, Goose	Yes
2023	Dazhou^3^	No	Yes#	No	No	No	—	Yes#
2023	Bazhong^2^	Yes	Yes	No	No	No	—	No
2023	Ziyang	Yes	Yes	No	No	No	—	Yes

* a for farm exposures, # for positive detections of H5 only.

### 3.5 Environmental monitoring

**Figure 6:**
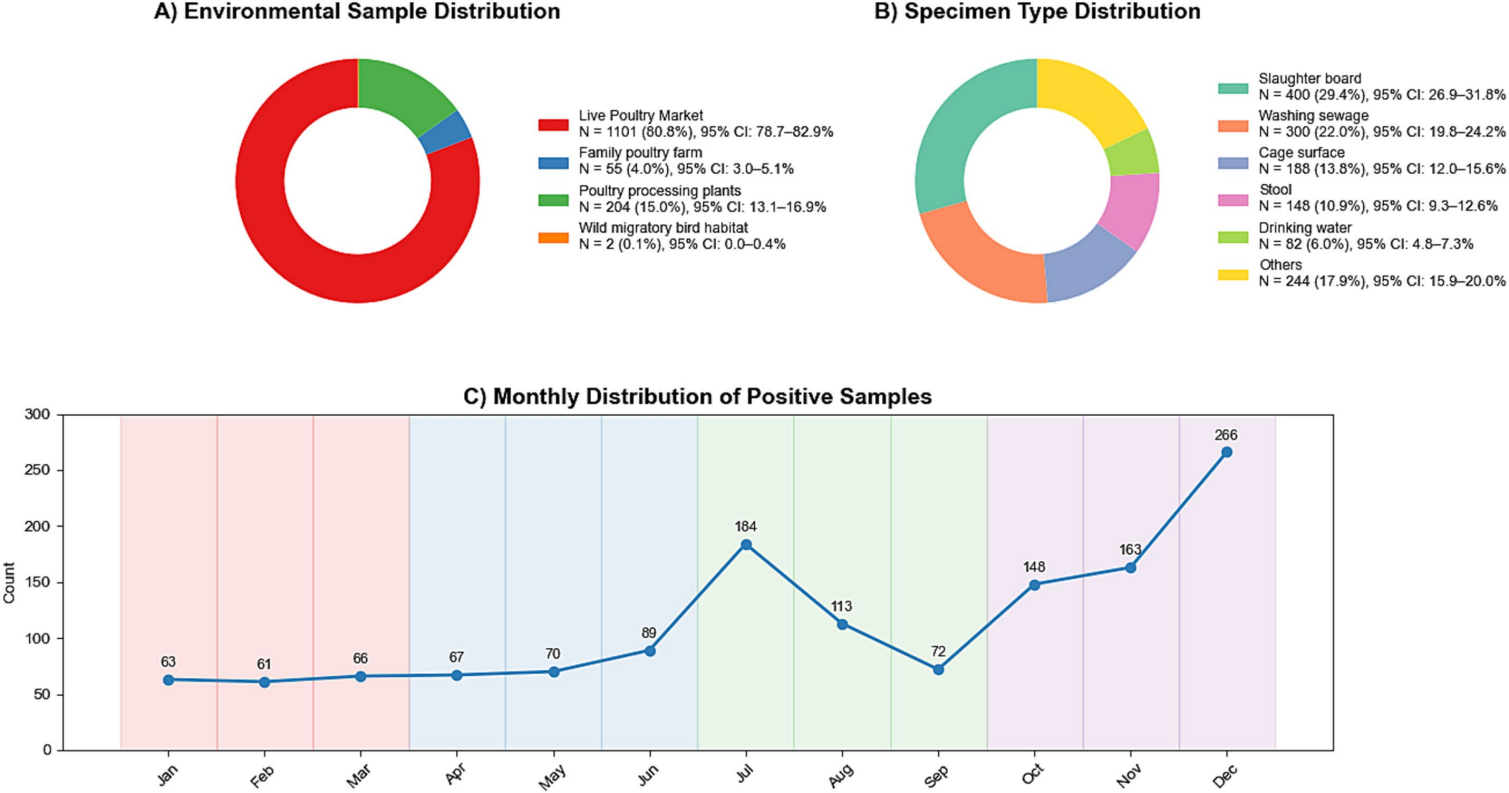
Pie chart of sample sources and locations for testing positive for subtype H5. **(A)** Environmental sample distribution; **(B)** Specimen type distribution; **(C)** Monthly distribution of positive samples.

Environmental monitoring in Sichuan Province is conducted in line with the Sichuan Provincial Avian Influenza Environmental Monitoring Program. Every city gathers a minimum of 10 poultry-related samples monthly. Sampling stations are designated in regions with elevated exposure concerns, including poultry drinking water, faeces, and areas of intense poultry operations. The gathered specimens are preserved at 4°C, dispatched to the laboratory within 48 h, and the viral nucleic acid assay is finalized within 1 week. Between 2019 and 2024, 14,397 external environmental samples were collected, of which 1,362 tested positives for the H5 subtype, resulting in a positivity rate of 9.46%. Figure 6 illustrates the monthly distribution of positive test results for the H5 subtype. The months with the highest number of H5 positive samples are July, October, and December. Figure 6 provide the sources of H5 positive samples, respectively. Among the monitored sites, the poultry slaughter board (400 instances) and the sewage used for washing poultry (300 cases) exhibited the highest number of positive samples; regarding sample sources, the live poultry market recorded the most positive samples (303 cases).

## 4 Discussion

The cases identified in Sichuan Province placed second, constituting 17.39% of all H5N6 cases. Prior research indicates that the death rate for human infections with H5N6 avian influenza can reach 55.4%, predominantly affecting older individuals, with a median age of around 51 years (9). Our study findings support this perspective. The case fatality rate for human infections with H5N6 avian influenza in Sichuan Province was 75%, with a median age of 54.5 years. The initial human infection strain in Sichuan Province in 2014 (A/Sichuan/1/2014) was a reassortant of H5N1 and H6N6, with its HA gene classified under Clade 2.3.4.4 (1). The HA gene of the strain predominant in Guangdong Province is classified within Clade 2.3.4.4, but the origin of the NA gene is intricate. In contrast to the strain in Sichuan Province, the strain in Jiangsu Province has a V100A mutation in the PB1-F2 protein (43, 44). The H5N6 strain isolated in early Europe (Germany A/duck/Germany/AR844/2007) has minimal pathogenicity, with its HA gene classified under Clade 2.2.1, distinct from the evolutionary lineage of highly pathogenic strains in Asia (45).

The primary route of transmission of AIVs often occurs in wild birds (46). AIVs and genetic pieces can sustain viral genetic variety via the dissemination of wild birds (47). Sichuan Province serves as a significant nexus in the migratory pathway of avian species in western China, boasting a diverse array of bird species. The China Bird Watching Record Center^1^ reports that Sichuan Province is the second most species-rich area in the nation, with 699 species documented. This establishes the conditions for the dissemination and genetic recombination of AIVs among various wild bird species (46). The capacity of viruses to traverse species boundaries is generally restricted, and AIVs cannot swiftly multiply in humans directly (48). Nonetheless, avian transmission may indirectly elevate the risk of human infection, particularly in regions exhibiting significant genetic variety of the virus, where variants capable of circumventing barriers may be more prone to emergence (49). Despite the seldom positive tests for the H5 subtype in specimens gathered from wild bird habitats in Sichuan Province, the unique ecological attributes may still be linked to the elevated occurrence of H5N6 infections. The virus’s genetic variety may be perpetually augmented by transmission by migrating birds, leading to Sichuan Province having one of the highest incidences of H5N6 case reports in the nation. Future collaborative ecological-epidemiological investigations are necessary to further substantiate the causal link.

Avian influenza is a secondary transmission pathway via poultry farms and associated transportation networks (50). The cumulative monthly case distribution exhibited a bimodal pattern in winter and summer, aligning with the H5 positivity distribution results seen in our external environment. The winter peak may result from low temperatures facilitating the prolonged survival of the avian influenza virus in external environments, such as feces, whereas the summer peak may stem from a decrease in poultry immunity under high-temperature and high-humidity conditions, hence triggering the epidemic (36, 51). Results from environmental monitoring indicate that live poultry markets are the primary source of avian influenza virus detection. Poultry slaughtering boards and washing wastewater are the two sample classes with the highest number of positive specimens, indicating a significantly elevated risk of viral transmission during poultry slaughtering and washing processes (52). The affirmative identification of cage surface and fecal samples indicates that the transportation of live poultry and fecal contamination remain significant transmission pathways, and inadequate sanitation of transport cages may result in cross-regional transmission (52). The prevalence of positives on family poultry farm and natural migratory bird habitats is comparatively low, maybe because to the limited size of breeding or inadequate monitoring of migratory bird habitats. Despite the low incidence of positive detections, the potential for farmed poultries to interact with wild birds may facilitate viral mutation and recombination (53).

The investigation’s findings indicated that contact with deceased poultry or their feces was the primary mode of infection for the case, succeeded by exposure to live poultry markets. The eastern and southern districts of Sichuan Province exhibit a substantial volume of poultry breeding and commerce, facilitating the maintenance and dissemination of the virus to a considerable degree (54). Temporary market closures in Guangdong, Shanghai, and other regions have markedly diminished the probability of H7N9 pandemic spread (55). The epidemic was contained after Guangdong halted live poultry trading during the Spring Festival in 2015, and the Xuhui District of Shanghai has not reported any human infection cases since the permanent closure of its live poultry market in 2013, demonstrating that prolonged closure can effectively impede the virus’s transmission (50, 56). Nevertheless, owing to inadequate law enforcement and the populace’s love for live poultry in Nanjing and other locales, illicit commerce has persisted despite several prohibitions, undermining the efficacy of preventative and control measures (57). This indicates that market closure regulations must be supplemented by rigorous law enforcement and public education initiatives. In rural regions, altering entrenched consumption patterns or constrained resources poses challenges for policy implementation. Future interventions in live poultry markets will necessitate a more adaptable approach (50, 55).

Chickens and ducks constituted the predominant species among the deceased poultry with whom the cases had contact, reflecting the prevalent breeding practices in Sichuan Province. The high-density breeding might have resulted in frequent interactions among birds, facilitating the fast transmission of the virus through feces, respiratory secretions, and other means (58). It is important to recognize that migrating birds may interact with the environments around farms throughout their migration paths, potentially introducing wild diseases to chicken populations. Should wild viruses exchange genetic material with AIVs in poultry, new strains potentially more transmissible to humans may emerge (46). Of the cases with positive specimens identified in the market, 85.7% (6/7) of the samples from domestic fowl were positive. This indicates that the virus might be spread indirectly via environmental contamination, necessitating an expansion of the monitoring parameters for the external environment in the future. In 2021, the market conditions for cases in Bazhong and Zigong were unexamined or the outcomes were indeterminate; nonetheless, the home specimens yielded positive findings, suggesting inadequate testing coverage. In 2022, a case in Nanchong City had no history of exposure; yet, house specimens tested positive, suggesting the existence of an unidentified transmission chain. In the future, it is essential to address surveillance deficiencies and explore the potential for asymptomatic poultry to harbor the virus.

Individuals with preexisting conditions, pregnant women, and other demographics are at elevated risk of severe illness and mortality due to zoonotic avian influenza (59). Epidemiological survey data from 16 cases in Sichuan Province indicated a significant prevalence of underlying disorders, with just 3 instances lacking such conditions, of which 2 were successfully treated. The cure rate for individuals without comorbidities was much superior to that of those with comorbidities. Individuals infected with H5N6 in Sichuan Province typically have a tendency to postpone medical intervention following the commencement of the illness. The box plot results indicate significant disparities in the time of treatment across various instances, implying the absence of a cohesive and standardized protocol to govern the commencement timing of antiviral therapy. This may occur due to their frequent misidentification as typical colds in the first phases of the illness. The mean duration from the beginning of symptoms to hospital admission was 4.6 days, surpassing the advised treatment duration for antiviral medications against influenza (60). The elevated case fatality rate of human H5N6 virus infections in Sichuan Province is mostly attributable to delayed diagnosis and treatment. The majority of patients reside in rural regions and initially pursue medical care at primary healthcare facilities or self-medicate, leading to lost chances for early intervention. Simultaneously, as human infections with avian influenza are few, medical institutions have limited exposure to such cases, resulting in a deficiency of experience in early detection. The duration from beginning to diagnosis is prolonged, hence hindering the prompt administration of anti-influenza medications.

During the 10-day monitoring period for close contact tracking, no abnormal symptoms were observed, and all respiratory specimens tested negative, indicating that the H5N6 avian influenza virus is not yet capable of human-to-human transmission, consistent with the characteristic difficulty AIVs have in overcoming the “bird-to-human” transmission barrier (9, 61). H5N6 strains exhibit a pronounced affinity for the *α*2-3 sialic acid receptor (SAα2-3Gal), prevalent in the respiratory and gastrointestinal systems of avian species (62). While several strains exhibit partial affinity for human-like α-2,6-linked sialic acid receptors *in vitro*, their binding rate remains markedly inferior to that of influenza viruses adapted to humans. In comparison to other avian influenza viruses (such as H7N9 and H5N1), H5N6 strains predominantly remain exclusive to avian receptors and seldom propagate in clusters (62, 63). Certain mutations indicate that H5N6 may possess the capability to adapt to certain animals. The E627K mutation in the PB2 gene can augment the virus’s replication capacity in mammalian cells, while the HA-T160A mutation may result in the elimination of glycosylation sites, hence enhancing the virus’s evasion of host protection (64, 65). The existing receptor binding range and gene mutation pattern collectively provide the constraints on human-to-human transmission, and the critical location (HA-Q226L/G228S) remains unmutated. Consequently, the immediate pandemic threat posed by the H5N6 virus is quite minimal (66, 67). The significant diversity of the virus and its strong connection to chicken markets will persist in necessitating enhanced viral surveillance and interdisciplinary collaboration to mitigate possible pandemic threats. Despite the virus’s now restricted transmissibility among individuals, ongoing surveillance of its genetic alterations is essential.

Currently, China lacks an avian influenza immunization initiative for high-risk populations. Nevertheless, several nations have previously used these techniques on an international scale. In 2024, Finland initially revealed intentions to immunize persons at occupational risk of avian influenza, including veterinarians and laboratory technicians (68). In 2025, Canada announced the acquisition of 500,000 doses of the human avian influenza vaccine, distributing them to high-risk populations, including laboratory personnel, close connections, and agricultural workers (69). These initiatives underscore the potential of vaccination as a crucial preventative measure for humans in the future, and tailored vaccination efforts for high-risk populations may also be executed in China thereafter.

The limitation of our analysis is that 16 cases insufficiently represent the whole risk profile of the province, necessitating the integration of additional epidemiological data about subtypes and locations. Considering our limited sample size, a non-significant *p*-value does not inherently indicate an absence of practical or therapeutic significance; rather, we must interpret these findings with care. Future research should integrate data from other locations to enhance statistical power and the generalizability of the findings. Nucleic acid testing has only been performed on environmental samples; research such as viral load measurement, genetic identification of isolates, and comparative study of viral sequences between poultry and human cases have not yet been done. Subsequent experiments may be undertaken to investigate the kinetics of viral transmission.

In the short term, we intend to enhance our findings by offering specific policy recommendations that might directly inform public health policy. We propose the implementation of advanced surveillance systems focused on regions with substantial chicken supply and closeness to live poultry markets, alongside systematic monitoring of wild bird populations and poultry farms, while also tracking human cases. These tools will facilitate the early detection of epidemics and inform targeted interventions. Furthermore, we endorse the development of explicit and succinct risk communication materials aimed at vulnerable populations and healthcare professionals, as well as the use of several communication channels to guarantee extensive distribution. We advocate for the creation of clinical management algorithms to assist healthcare professionals in the timely identification and treatment of H5N6 infections, highlighting the importance of early antiviral therapy for high-risk populations and the use of extracorporeal membrane oxygenation (ECMO) in severe instances. Prolonged investigation for forthcoming developments, we advocate for the establishment of targeted vaccination initiatives for high-risk populations, regular disinfection procedures for poultry farms and live poultry markets, and cost-effectiveness assessments to identify the most economically viable strategies for preventing outbreaks and alleviating social repercussions. The integration of human and animal monitoring systems is still constrained, and mechanisms for direct data sharing with agricultural authorities have not been completely realized. In light of the rise in instances, further study will incorporate additional socio-demographic characteristics, socio-economic status, and health literacy levels. We also promote the enhancement of interdepartmental data connectivity to bolster early warning systems and augment the surveillance of avian influenza under the “One Health” plan.

## 5 Conclusion

Sichuan Province has a significant prevalence of H5N6, and the cases with elevated fatality rates are of considerable concern. The infection risk of avian influenza in the province is mostly associated with exposure to live poultry markets, contact with deceased poultry, and environmental contamination, with chickens and ducks serving as the principal hosts. The province serves as a migratory habitat for avian species. In the future, it may be essential to disrupt the transmission chain through comprehensive interventions involving “wild birds-live poultry trading markets-breeding areas-households,” and to address the threat of potential avian influenza variants by enhancing environmental monitoring and virus traceability research.

## Data Availability

The raw data supporting the conclusions of this article will be made available by the authors, without undue reservation.

## Footnotes

^1^ http://www.birdreport.cn
